# How the ghrelin receptor recognizes the acyl-modified orexigenic hormone

**DOI:** 10.3389/fnmol.2025.1549366

**Published:** 2025-04-07

**Authors:** Yuki Shiimura, Masayasu Kojima, Takahiro Sato

**Affiliations:** ^1^Division of Molecular Genetics, Institute of Life Science, Kurume University, Fukuoka, Japan; ^2^Department of Cell Biology, Graduate School of Medicine, Kyoto University, Kyoto, Japan

**Keywords:** ghrelin, ghrelin receptor (GHS-R1a), GPCR, structural biochemistry, orexigenic hormone

## Abstract

Ghrelin, discovered in 1999 as an endogenous ligand of the growth hormone secretagogue receptor (now known as the ghrelin receptor), is a peptide hormone with diverse physiological activities, such as stimulation of growth hormone release, increased appetite, fat accumulation, thermoregulation, and cardioprotection. As a distinctive feature, ghrelin needs to undergo octanoylation, a specific acyl modification, to exert its biological activities. Although the ghrelin receptor specifically recognizes this modification, the underlying molecular mechanism had remained unclear for decades. Recent advancements in structural biology have facilitated the elucidation of this recognition mechanism 25 years after ghrelin’s discovery. This review highlights the structural basis of ghrelin octanoylation, particularly emphasizing the mechanism by which the ghrelin receptor recognizes this acyl-modified hormone.

## Introduction

In 1980, a Met-enkephalin-like pentapeptide was found to exhibited growth hormone (GH)-releasing activity (Bowers et al., 1980). Although the GH-releasing effect was extremely weak and could only be observed *in vitro*, it specifically promoted GH release without affecting the secretion of other anterior pituitary hormones, including luteinizing hormone, follicle-stimulating hormone, thyroid-stimulating hormone, and prolactin. This discovery was followed by the development of more potent peptide ligands, such as GH-releasing peptide-6, in the early 1980s to enhance GH-releasing activity *in vivo* (Bowers et al., 1984; Momany et al., 1984). In 1993, the first small-molecule ligands, L-692,429 and its superior derivative, L-163,191 (MK-0677), were developed, further accelerating research in this field (Table 1) (Smith et al., 1993; Cheng et al., 1993; Patchett et al., 1995). Meanwhile, the GH-releasing hormone (GHRH), secreted from the hypothalamus, was known to stimulate GH secretion from the pituitary gland. GHRH activates the GHRH receptor (GHRH-R) by stimulating intracellular cAMP levels, resulting in GH secretion (Mayo, 1992). Although GHRH-R couples with the G_s_ protein, the addition of MK-0677 to rat primary cultured pituitary cells increased intracellular Ca^2+^ levels, suggesting the existence of a second G protein-coupled receptor (GPCR) that couples with the G_q_ protein, thereby promoting GH secretion through a pathway distinct from that of GHRH-R (Patchett et al., 1995). Hence, the above-discussed peptides and small-molecule agonists capable of enhancing GH secretion are referred to as GH secretagogues (GHSs). In 1996, the second receptor, the GHS receptor 1a (GHS-R1a), was identified using MK-0677 and *Xenopus* oocytes injected with polyadenylated [poly(A)^+^] RNA derived from the swine pituitary (Howard et al., 1996). Subsequently, DNA sequence analysis identified GHS-R1a as a GPCR, and *in situ* hybridization revealed that it is expressed in the pituitary gland, as well as in the hypothalamus and hippocampus (Bennett et al., 1997; Guan et al., 1997). However, the endogenous ligand of GHS-R1a remains unknown, which has fueled intense interest in the deorphanization of this receptor.

**Table 1:** The history of the discovery of GHS and ghrelin.

Ligand	Sequence or chemical structure	Feature	References
Met-enkephalin analog	Tyr-^D^Trp-Gly-Phe-Met-NH_2_ Tyr-^D^Phe-Gly-Phe-Met-NH_2_	First GHS	Bowers et al. (1980)
GHRP-6	His-^D^Trp-Ala-Trp-^D^Phe-Lys-NH_2_	GH secretory activity *in vivo*	Bowers et al. (1984) and Momany et al. (1984)
L-692,429	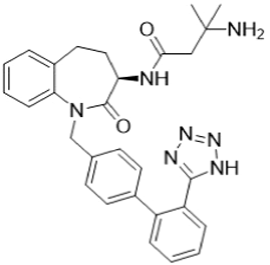	First non-peptide GHS	Smith et al. (1993) and Cheng et al. (1993)
L-163,191 (MK-0677)	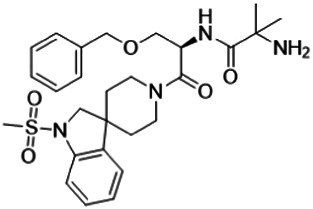	Orally active GHS	Patchett et al. (1995)
Ghrelin (Rat)	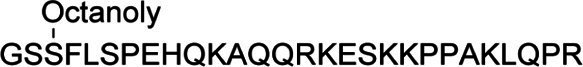	Endogenous ligand	Kojima et al. (1999)

In 1999, in a notable discovery, the endogenous ligand of the centrally localized GHS-R1a was identified in peripheral tissue (Kojima et al., 1999). The research group led by Kojima and Kangawa was inspired by the fact that motilin, the endogenous ligand for GPR38 (the motilin receptor), which shares high amino acid homology with GHS-R1a, is secreted from the gastrointestinal tract. This inspired their investigation into identifying the endogenous ligand of GHS-R1a in gastrointestinal tissues extracts (Feighner et al., 1999). Ghrelin has been successfully isolated from the rat stomachs of rats as an endogenous ligand of GHS-R1a. Ghrelin exhibits a highly intriguing structural feature: octanoylation (C8:0) of the serine residue at position 3 (Ser3) from the N-terminus is essential for activation of GHS-R1a (hereafter referred to as the ghrelin receptor). Although 25 years have passed since the discovery of ghrelin, no other hormones requiring fatty acid modification for activation have been identified. In other words, the ghrelin receptor is the only GPCR that discriminates between the acyl modifications of peptide hormones. However, the molecular mechanism underlying this recognition is yet to be elucidated, a major question in ghrelin research. Recent advances in structural biology techniques, including X-ray crystallography and single-particle cryo-electron microscopy (Cryo-EM), have provided a clearer understanding of the ghrelin receptor (Shiimura et al., 2020; Wang et al., 2021; Liu et al., 2021; Qin et al., 2022; Shiimura et al., 2025), revealing that the ligand-binding pocket of the ghrelin receptor features a unique architecture referred to as a “bifurcated pocket,” comprising two distinct cavities. Furthermore, acyl-ghrelin was found to bind to the ghrelin receptor by effectively utilizing this “bifurcated pocket.”

In addition to promoting GH secretion (Kojima et al., 1999), the ghrelin-ghrelin receptor system participates in appetite stimulation, body temperature regulation, and cardioprotection (Nakazato et al., 2001; Sato et al., 2021; Nagaya et al., 2001). Accordingly, this system could be developed for various clinical applications, including the treatment of familial short stature, cancer cachexia, eating disorders, and sarcopenia (Kojima and Kangawa, 2005). Structural information on the ghrelin receptor is expected to facilitate the discovery of drugs targeting these diseases.

### Acyl-modified hormone: ghrelin

Ghrelin, a peptide hormone isolated and purified from the rat stomach in 1999, is a endogenous ligand of the GHS receptor (now referred to as the ghrelin receptor) (Kojima et al., 1999). The name “ghrelin” is derived from the words “growth hormone release” and the Proto-Indo-European root “*ghre*,” meaning “to grow,” reflecting its role in stimulating GH secretion. Ghrelin is primarily synthesized in the gastrointestinal tract, particularly in the stomach and the duodenum (Hosoda et al., 2000). Ghrelin is also produced in the central nervous system, including the hypothalamus and pituitary gland, although levels in these tissues are minimal. The stomach contains an abundance of ghrelin, predominantly secreted by X/A-like cells.

**Figure 1:**
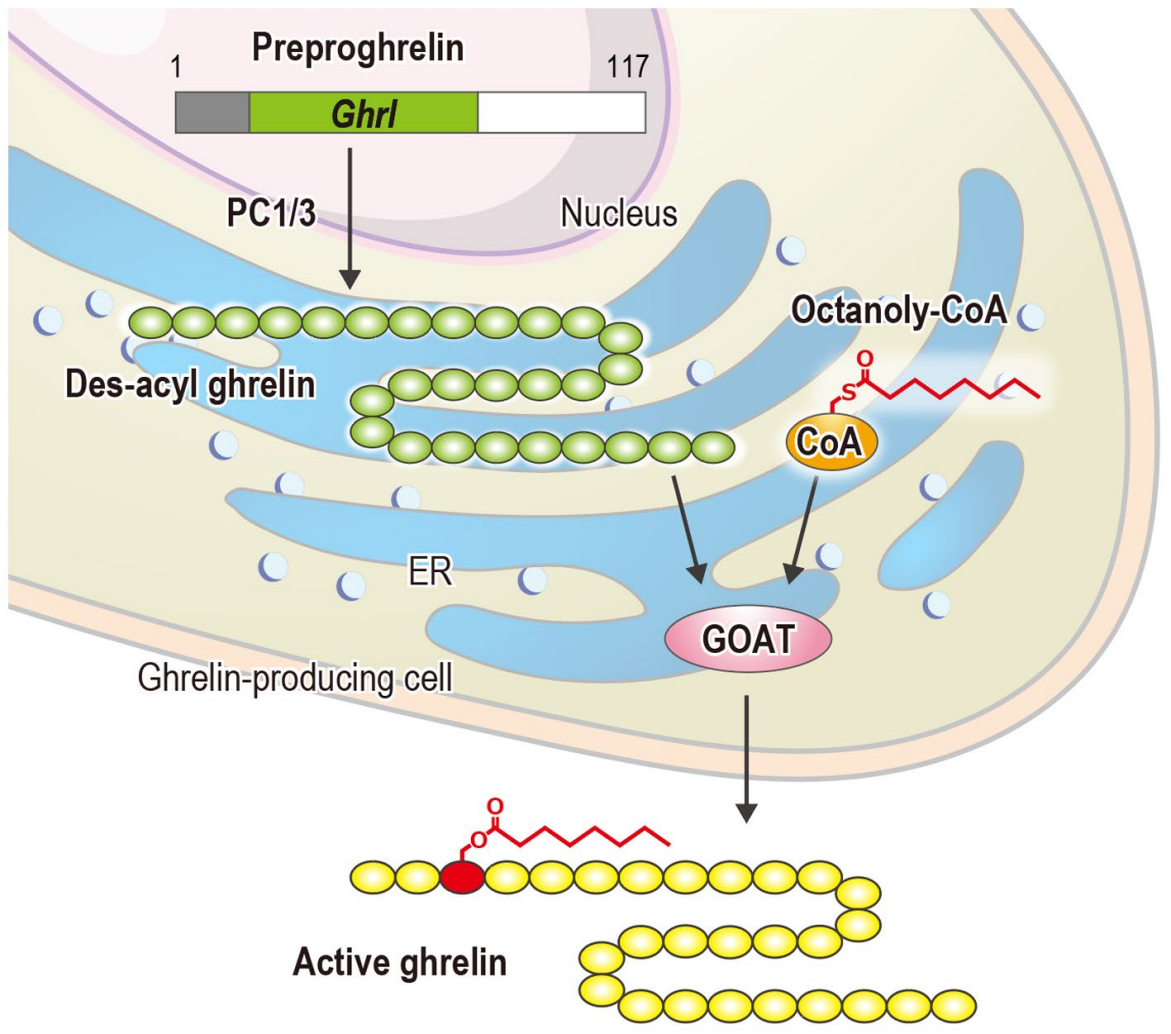
Biosynthesis of ghrelin: PC 1/3 and ER refer to prohormone convertase 1/3 and the endoplasmic reticulum, respectively.

The human ghrelin gene is located on chromosome 3p25-26. After translation, human ghrelin becomes a 117-amino acid preprohormone, which undergoes cleavage by prohormone convertase 1/3 to produce a final 28-amino acid peptide (Zhu et al., 2006). This unmodified peptide, des-acyl ghrelin, cannot bind to the ghrelin receptor (Shiimura et al., 2020). Subsequently, in the endoplasmic reticulum membrane, ghrelin *O*-acyltransferase (GOAT; also known as MBOAT4), a member of the membrane-bound *O*-acyltransferase (MBOAT) family, utilizes octanoyl-CoA as a donor to attach octanoic acid to Ser3, converting it into active ghrelin, which is then secreted into the bloodstream (Figure 1) (Gutierrez et al., 2008; Yang et al., 2008). Notably, GOAT is the only enzyme in the MBOAT family that catalyzes substrate modification using medium-chain fatty acids (Tuladhar and Lum, 2015). However, the molecular mechanisms underlying this unique process remain elusive. Further studies are required to clarify the structural and functional bases of this selective catalysis.

In rats, gastric concentrations of active and total ghrelin were 377 and 1780 fmol/mg wet tissue, respectively (Hosoda et al., 2000). In human plasma, active ghrelin levels typically range from 10 to 20 fmol/mL, whereas those of total ghrelin range from 150 to 250 fmol/mL, with approximately 10% undergoing acylation, a modification crucial for its biological activity (Cummings et al., 2001; Tschöp et al., 2001). In tissues, the fatty acid modification of ghrelin predominantly involves octanoic acid (C8:0), followed by decanoic acid (C10:0) and hexanoic acid (C6:0), and is limited to saturated medium-chain fatty acids (Hosoda et al., 2003). Intriguingly, when rats were fed a diet containing heptanoic acid (C7:0), a medium-chain fatty acid that is not biosynthesized in the body, ghrelin modified with heptanoic acid was detected in their blood (Nishi et al., 2005). These findings suggest that dietary saturated medium-chain fatty acids can be directly utilized for ghrelin modification. Ghrelin has been extracted from various vertebrates, and its amino acid sequences have been identified (Kaiya et al., 2004; Kaiya et al., 2003; Kaiya et al., 2003; Kaiya et al., 2003; Kaiya et al., 2001; Kaiya et al., 2002). Notably, the N-terminal 10 amino acids are highly conserved among mammals, whereas the N-terminal 7 amino acids exhibit strong conservation across vertebrates, suggesting that this region serves as the functional core of ghrelin. Ser3, critical for acylation-mediated, receptor activation, is preserved in nearly all species except for bullfrogs, where it is replaced by threonine (Thr3) (Kaiya et al., 2001). Remarkably, Thr3 retains its ability to undergo acylation, highlighting the functional significance of this modification.

Shortening the fatty acid chain length of rat ghrelin was reported to gradually increase the EC_50_ (half maximal effective concentration) for receptor activation by 10-fold with hexanoyl (C6:0), 200-fold with butyryl (C4:0), and 500-fold with acetyl (C2:0) (Matsumoto et al., 2001). Conversely, elongating the palmitoyl chain (C16:0) did not substantially alter EC_50_. These findings emphasize the importance of medium- or longer-chain fatty acids for ghrelin receptor activation *in vitro* while highlighting the remarkable tolerance of the receptor for long-chain fatty acids.

### Distribution of the ghrelin receptor

In rat tissues, the ghrelin receptor is abundantly expressed in the hypothalamic arcuate nucleus, a key center for feeding regulation (Korbonits et al., 2004; Van Der Lely et al., 2004; Tannenbaum et al., 1998; Georgi et al., 1999). It is also expressed in the suprachiasmatic nucleus, anteroventral tegmental, preoptic, paraventricular, and nucleus accumbens within the hypothalamus (Howard et al., 1996; Guan et al., 1997). In brain regions other than the hypothalamus, ghrelin receptor expression was detected in the CA2 and CA3 regions of the hippocampus, substantia nigra, ventral tegmental area, dorsal raphe nucleus, and median raphe nucleus, indicating its potential role in learning and memory (Guan et al., 1997; Muccioli et al., 1998; Katayama et al., 2000). Additionally, the ghrelin receptor is abundantly expressed in somatotrophs of the pituitary gland, supporting the role of ghrelin in regulating GH release.

Although the ghrelin receptor is predominantly expressed in the central nervous system, a broad expression pattern of the gene encoding the ghrelin receptor is observed in various peripheral tissues, including the thyroid, pancreas, spleen, myocardium, adrenal gland, testis, ovary, stomach, and intestinal neurons (Gnanapavan et al., 2002; Gaytan et al., 2004; Gaytan et al., 2003; Gaytan et al., 2005; Dass et al., 2003; Kageyama et al., 2005; Shuto et al., 2001). This peripheral distribution of the ghrelin receptor supports the concept that ghrelin functions beyond the regulation of energy metabolism and GH secretion.

In human tissues, the ghrelin receptor mRNA has been detected in the normal anterior pituitary gland, pituitary adenoma, hypothalamus, and hippocampus. Additionally, its mRNA expression was observed in peripheral tissues such as the adrenal cortex, testis, pancreas, heart, and lung (Mckee et al., 1997; Yokote et al., 1998). Gene expression of the ghrelin receptor was identified in pituitary adenomas, neoplastic thyroid tissue, breast cancer, prostate cancer cell lines, and ovarian tumors (Gaytan et al., 2005; Korbonits et al., 2001; Barlier et al., 1999; Skinner et al., 1998; Korbonits et al., 1998; Adams et al., 1998; Kim et al., 2001; Volante et al., 2003; Cassoni et al., 2001; Cassoni et al., 2004; Jeffery et al., 2002). These findings suggest that ghrelin plays a role in regulating cancer cell growth in certain tumors (Jeffery et al., 2003).

The expression of ghrelin receptor mRNA is suppressed by GH but stimulated by GHRH (Bennett et al., 1997; Kamegai et al., 1998; Horikawa and Tachibana, 2000; Nass et al., 2000; Kineman et al., 1999). In the hypothalamic arcuate nucleus, leptin, known for its antagonistic and anorexigenic effects on ghrelin, was found to reduce ghrelin receptor expression (Nogueiras et al., 2004). In the pituitary gland, levels of ghrelin receptor mRNA were suppressed by insulin-like growth factor-I (Kamegai et al., 2005; Zhou et al., 1997; Peng et al., 2001; Wallenius et al., 2001). Moreover, thyroid hormones, glucocorticoids, and sex steroids reportedly regulate ghrelin receptor mRNA expression (Petersenn et al., 2001; Kaji et al., 2001; Kamegai et al., 2001; Tamura et al., 2000; Thomas et al., 2000; Kamegai et al., 1999).

The ghrelin receptor also has a splice variant known as GHS-R1b, comprising only five transmembrane helices. Expression of GHS-R1b has been widely detected in tissues, including the skin, myocardium, pituitary, thyroid, pancreas, ileum, colon, somatic tachycardia tumors, liver, breast, spleen, duodenum, placenta, lung, adrenal gland, buccal mucosa, stomach, lymph nodes, atrial lymphocytes, and kidneys. However, GHS-R1b does not function as a receptor for ghrelin (Gnanapavan et al., 2002).

### Overall structure and ligand-binding pocket of the ghrelin receptor

**Figure 2:**
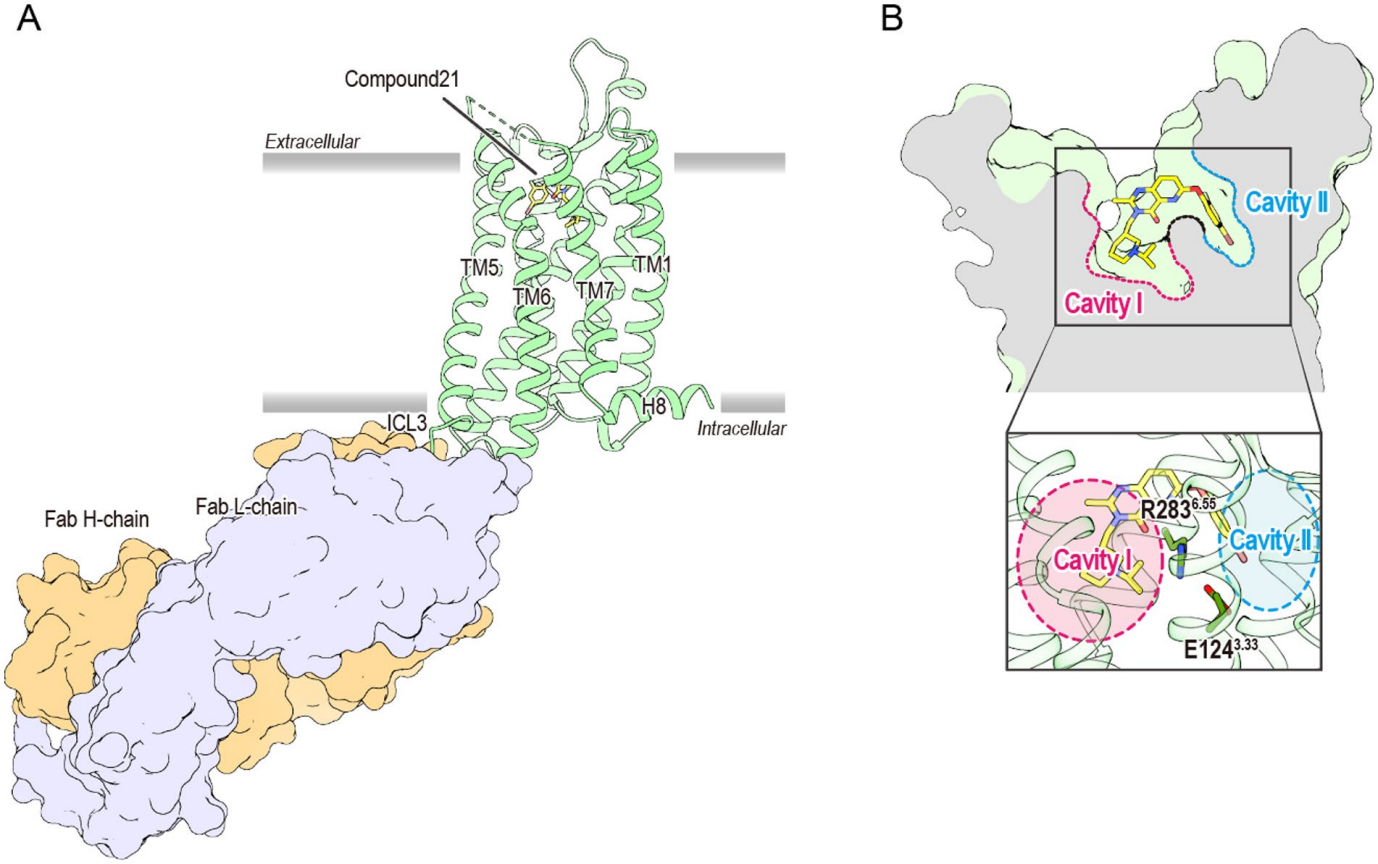
**(A)** The overall structure of the antagonist-bound ghrelin receptor in complex with its antibody (PDB ID: 6KO5). The ghrelin receptor is shown in green, and the antagonist in yellow. The ghrelin receptor antibody, Fab L chain, and H chain are shown in orange and lavender, respectively. The dashed line between TM6 and TM7 indicates disordered region. TM and H indicate the transmembrane and helix, respectively, while ICL refers to the intracellular loop. **(B)** Cross-sectional view of the ligand-binding pocket of the ghrelin receptor. Cavities I and II are shown in magenta and cyan, respectively. The two cavities are separated by a salt bridge formed between Glu124^3.33^ and Arg283^6.55^.

For over two decades, the mechanism through which the ghrelin receptor recognizes the octanoylation of ghrelin remained unresolved. However, recent advances in structural biology have provided detailed insights into the underlying molecular mechanisms. X-ray crystallography established the first three-dimensional structure of the ghrelin receptor in an inactive conformation using its high-affinity antagonist Compound 21 and a ghrelin receptor-specific antibody in 2020 (Figure 2A) (Shiimura et al., 2020). Compound 21 is a modified derivative of YIL781, a commercially available ghrelin receptor antagonist. The Ki values of YIL781 and Compound 21 are 4.0 nM and 0.01 nM, respectively, indicating that Compound 21 has 400 times higher binding affinity (Hanrahan et al., 2012). The anti-ghrelin receptor antibody not only functions as a binder to facilitate the crystallization of the ghrelin receptor but also contributes to its stabilization. In general, membrane proteins such as GPCRs are known to be difficult to crystallize because they are mostly surrounded by detergent micelles upon solubilization. Soluble proteins like antibodies, which remain exposed beyond the detergent micelles, aid in crystal formation. Furthermore, the anti-ghrelin receptor antibody developed in this study improved the thermal stability of the ghrelin receptor from 57.5°C to approximately 62.0°C. These pieces of evidence suggest that these techniques played a crucial role in determining the first structure of the ghrelin receptor.

The ghrelin receptor adopts a typical GPCR architecture, featuring seven transmembrane helices (TM1–7) and a short amphipathic helix8 on the intracellular side. However, the ligand-binding pocket exhibited two unique structural features that were not observed in previously determined GPCRs.

First, the ligand-binding pocket is split into two distinct regions, forming a “bifurcated pocket.” Within this pocket, a salt bridge between Glu124^3.33^ and Arg283^6.55^ [superscripts denote the Ballesteros–Weinstein numbering system (Ballesteros and Weinstein, 1995)] divides the ligand-binding pocket into two cavities: a relatively large Cavity I and a smaller Cavity II (Figure 2B). Compound 21 straddles this salt bridge, occupying both cavities and stabilizing the bifurcated pocket.

**Figure 3:**
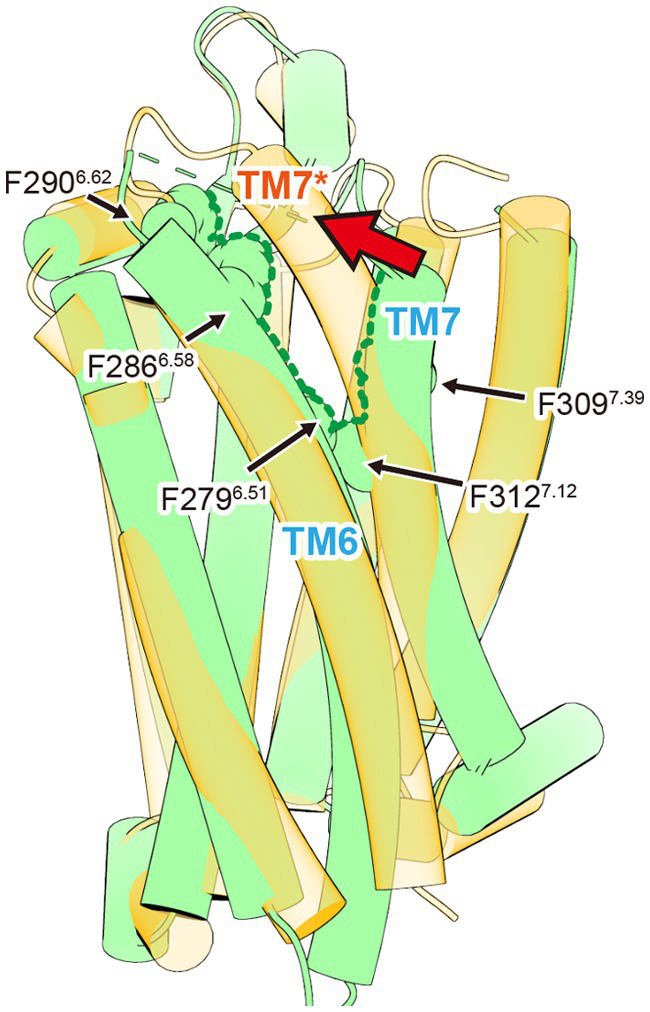
The crevasse observed in the inactive ghrelin receptor. The inactive and active ghrelin receptors are shown in green and orange, respectively. The green dished line indicates the crevasse in the inactive ghrelin receptor. The asterisk marks TM7 of active ghrelin receptor. Upon ghrelin receptor activation, the crevasse disappears as TM7 shifts toward TM6. This TM7 shift is indicated by the red arrow.

The second distinctive feature is a gap-like structure called “crevasse,” observed between TM6 and TM7. Crevasse is characterized by a cluster of hydrophobic phenylalanine residues, including Phe279^6.51^, Phe286^6.58^, Phe290^6.62^, Phe309^7.39^, and Phe312^7.42^ (Figure 3). The hydrophobicity of the phenylalanine cluster in crevasse, along with the ability of fatty acids longer than octanoic acid (C8:0), such as lauric acid (C12:0) and palmitic acid (C16:0), to activate the ghrelin receptor, initially led to the hypothesis that the fatty acid chain of ghrelin extends through this crevasse into the cell membrane (Matsumoto et al., 2001). Comparisons with lipid-binding GPCRs, including S1P1 and CB1, further supported this idea (Hua et al., 2017; Hanson et al., 2012). However, this hypothesis was refuted following the determination of the structure of the ghrelin-bound ghrelin receptor, revealing an alternative binding mode. The structural insights obtained so far do not explain why the ghrelin receptor can accommodate long-chain acylated ghrelin. Further analysis is required to elucidate this mechanism.

### Structural insights into ghrelin recognition by the ghrelin receptor

Following the publication of the inactive ghrelin receptor structure in 2020, a series of ghrelin receptor structures were determined using cryo-EM and subsequently reported (Wang et al., 2021; Liu et al., 2021; Qin et al., 2022; Shiimura et al., 2025). These structures represented the ghrelin receptor in complex with different G proteins (Table 2). To date, the binding modes of various ligand types, including the endogenous ligand ghrelin, the peptide agonist, the small-molecule agonist, the antagonist, and the inverse agonist, have been elucidated. These findings enrich our understanding of the structural biology of the ghrelin receptor, particularly its ligand recognition and activation mechanisms. For example, structural studies have shown that all ghrelin receptor agonists, regardless of ligand type, are accommodated within the “bifurcated pocket,” similar to Compound 21. Additionally, agonist binding is suggested to push Arg283^6.55^ downward, a residue critical for forming the “bifurcated pocket,” thereby shifting the receptor into its active conformation (Sakai et al., 2024). A particularly significant finding is that the ghrelin-bound forms provide detailed insights into the recognition of acyl-modified ghrelin.

**Table 2:** Reported ghrelin receptor structures.

PDB ID	State	Ligand	Coupling	Method	References
6KO5	inactive	Compound21	-	X-ray	Shiimura et al. (2020)
7F9Y	active	GHRP-6	G_q_	Cryo-EM	Wang et al. (2021)
7F9Z	active	Ghrelin	G_q_	Cryo-EM	
7NA7	active	Ghrelin-27	G_i_	Cryo-EM	Liu et al. (2021)
7NA8	active	MK-0677	G_i_	Cryo-EM	
7W2Z	active	Ghrelin	G_o_	Cryo-EM	Qin et al. (2022)
8F83	inactive	PF-05190457	-	X-ray	
8JSR	active	Anamorelin	G_q_	Cryo-EM	Shiimura et al. (2025)

Although G_q_, G_i_, or G_o_ protein complexes have been determined in these structures, the binding modes of ghrelin show minimal differences (Wang et al., 2021; Liu et al., 2021; Qin et al., 2022; Shiimura et al., 2025). The ghrelin receptor ingeniously utilizes its bifurcated pocket to bind ghrelin. Cavity I of the ghrelin receptor, which is relatively hydrophilic, contains four polar amino acids, including Asp99^2.60^ and Arg102^2.63^, in addition to the salt bridge. This cavity accommodates the peptide portion of ghrelin, with Asp99^2.60^ and Arg102^2.63^ interacting with Ser2 of ghrelin. Conversely, Cavity II, apart from the salt bridge, is highly hydrophobic and devoid of polar amino acids. This cavity accommodates the octanoyl group of ghrelin. Although the electron density at the distal end of the octanoyl group was not visible in the three structures, it undoubtedly resides within Cavity II. Moreover, the crevasse observed in the inactive structure was absent in the active form, because TM7 shifted toward TM6 after agonist binding (Figure 3).

### Structural comparison with the motilin receptor

**Figure 4:**
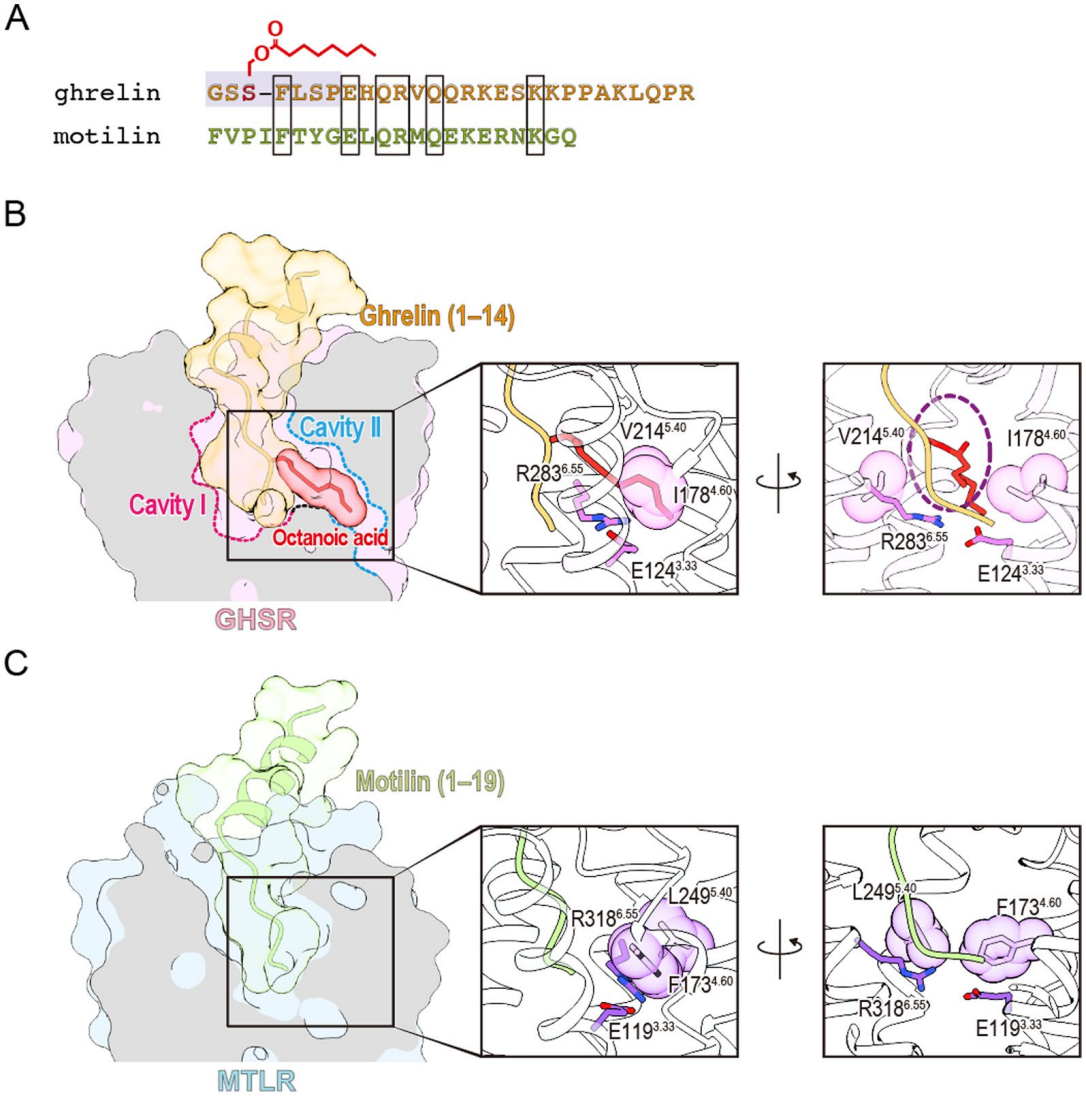
**(A)** Sequence comparison of ghrelin and motilin. The active center of ghrelin is highlighted in gray. Amino acids shared between the two peptides are enclosed in rectangles. **(B)** The ligand-binding pocket of the ghrelin-bound ghrelin receptor (PDB ID: 7F9Z). The ghrelin receptor, the peptide portion of ghrelin, and the octanoyl group of ghrelin are shown in pink, orange, and red, respectively. A dashed purple circle indicates the space accommodating the octanoyl group between Ile178^4.60^ and Val214^5.40^. **(C)** The ligand-binding pocket of the motilin-bound motilin receptor (PDB ID: 8IBV). The motilin receptor and motilin are shown in light blue and green, respectively.

Motilin is a 22-amino acid peptide hormone secreted from the upper gastrointestinal tract (Brown et al., 1971). It induces gastrointestinal motility by acting on motilin receptor, which are widely expressed in the gastric antrum, duodenum, colon, and rectum (Feighner et al., 1999; Miller et al., 2000). In recent years, the structure of the motilin-bound motilin receptor-G_q_ protein complex has been determined (You et al., 2023). Ghrelin and motilin receptors, along with the neuromedin U, neurotensin, and GPR38 receptors, form a cluster known as the ghrelin receptor family (Kojima and Kangawa, 2005). The amino acid sequence homology between the ghrelin and motilin receptors is strikingly high, reaching 52% overall and 86% in their transmembrane regions. Despite 27% sequence similarity between the two peptides, their N-terminal 7 amino acids, which are critical for ghrelin receptor activity display almost no similarity (Figure 4A). In addition, motilin lacks fatty acid modifications, making it incapable of binding to the ghrelin receptor. However, the opposing polar amino acids that divide the ligand-binding pocket into two cavities in the ghrelin receptor are conserved in the motilin receptor as Glu127^3.33^ and Arg337^6.55^. These residues also form a salt bridge in the ligand-binding pocket of the motilin receptor, similar to their role in the ghrelin receptor. Interestingly, the cavity corresponding to Cavity II in the ghrelin receptor is closed in the motilin receptor, and this closure is primarily attributed to Phe173^4.60^ and Leu249^5.40^ in the motilin receptor, corresponding to Ile178^4.60^ and Val214^5.40^ in the ghrelin receptor (Figures 4B,C). The bulkier Phe173^4.60^ and the inward orientation of Leu249^5.40^ effectively occlude Cavity II of the motilin receptor and prevent ligand penetration. This structural difference largely explains the inability of ghrelin to bind to the motilin receptor. Another factor contributing to Cavity II closure is the positioning of Met^5.39^. In the ghrelin receptor-G_q_ complex, Met213^5.39^ is oriented upward, allowing the accommodation of the octanoyl group (Wang et al., 2021). Conversely, Met248^5.39^ in the motilin receptor-G_q_ complex is directed toward Phe173^4.60^, further occluding Cavity II. Notably, this upward shift in Met213^5.39^ is specific to the ghrelin receptor-G_q_ complex and is absent in the ghrelin receptor-G_o_ and ghrelin receptor-G_i_ complexes (Liu et al., 2021; Qin et al., 2022). The electron density map supporting this Met213^5.39^ shift in the ghrelin receptor-G_q_ complex is incomplete and warrants cautious interpretation. Additionally, the phenylalanine-rich region named “crevasse” in the inactive structure of the ghrelin receptor is absent in the motilin receptor (Shiimura et al., 2020; You et al., 2023). In particular, Phe286^6.58^ and Phe290^6.62^, located at the entrance of the ligand-binding pocket, are thought to contribute to the accessibility of the acyl-modified peptide through their hydrophobic properties (You et al., 2023). However, this mechanism requires further investigation.

## Discussion

Structural analyses of the ghrelin receptor, enabled by advancements in structural biology techniques such as X-ray crystallography and cryo-EM, have provided detailed insights into the molecular mechanisms underlying the recognition of active ghrelin by its receptor. Given the diverse physiological roles of ghrelin, the ghrelin-ghrelin receptor system is expected to become a promising therapeutic target for various diseases related to metabolic disorders. Currently, the only ghrelin receptor-targeting drug approved in Japan is anamorelin, an agonist used to treat cancer cachexia. Cancer cachexia is a progressive syndrome characterized by weight loss, skeletal muscle wasting, inflammation, anorexia, and metabolic abnormalities. It affects approximately 50 to 80% of cancer patients and is associated with about 20% of cancer-related deaths (Argilés et al., 2014). Anamorelin is the first therapeutic drug in this field and holds the potential to improve the mortality rate and quality of life of cancer patients. In addition, recent studies have shown that ghrelin administration increases the cardiac output in patients with left ventricular dysfunction without severe adverse effects, highlighting the expanded therapeutic potential of this system (Lund et al., 2023). Building on these recent studies, the growing availability of structural insights is expected to substantially advance drug discovery efforts targeting the ghrelin receptor.
